# APPROACHES TO ITERATIVE ALGORITHMS FOR SOLVING NONLINEAR EQUATIONS WITH AN APPLICATION IN TOMOGRAPHIC ABSORPTION SPECTROSCOPY

**Published:** 2024-05-14

**Authors:** FRANCISCO J. ARAGÓN-ARTACHO, WEIWEI CAI, YAIR CENSOR, AVIV GIBALI, CHONGYUAN SHUI, DAVID TORREGROSA-BELÉN

**Affiliations:** 1 Department of Mathematics, University of Alicante, Alicante, Spain; 2 Department of Mechanical Engineering, Shanghai Jiao Tong University, Shanghai, China; 3 Department of Mathematics, University of Haifa, Haifa, Israel; 4 Applied Mathematics Department, HIT Holon Institute of Technology, Holon, Israel

**Keywords:** Nonlinear equations, common fixed point, cyclic sequential algorithm, tomographic absorption spectroscopy, alternating fixed points algorithm, descent pairs algorithm, superiorization, 35Q99, 47J05, 65K05, 90C30

## Abstract

In this paper we propose an approach for solving systems of nonlinear equations without computing function derivatives. Motivated by the application area of tomographic absorption spectroscopy, which is a highly-nonlinear problem with variables coupling, we consider a situation where straightforward translation to a fixed point problem is not possible because the operators that represent the relevant systems of nonlinear equations are not self-mappings, i.e., they operate between spaces of different dimensions.

To overcome this difficulty we suggest an “alternating common fixed points algorithm” that acts alternatingly on the different vector variables. This approach translates the original problem to a common fixed point problem for which iterative algorithms are abound and exhibits a viable alternative to translation to an optimization problem, which usually requires derivatives information. However, to apply any of these iterative algorithms requires to ascertain the conditions that appear in their convergence theorems.

To circumvent the need to verify conditions for convergence, we propose and motivate a derivative-free algorithm that better suits the tomographic absorption spectroscopy problem at hand and is even further improved by applying to it the superiorization approach. This is presented along with experimental results that demonstrate our approach.

## 1. INTRODUCTION

Solving systems of nonlinear equations has long been and still is a fundamental problem in mathematics with countless real-world applications that demand efficient methods to accomplish the task, see, e.g., the books by Ortega and Rheinboldt [36] and by Rheinboldt [37]. A Google Scholar search on this topic returns, not surprisingly, numerous entries. A central approach is based on transforming the systems of nonlinear equations to an optimization problem and using methods from that field, mainly methods for unconstrained minimization which are commonly geared toward convex functions, see, e.g., Boyd and Vandenberghe’s book [4].

In practical situations it is, however, often the case that no derivatives of the functions that comprise the system of equations are known and even if derivatives exist, they are not calculable. This hinders the applicability of minimization methods and a variety of heuristics have been developed such as, for example, the simulated annealing method, consult, e.g., the information on the auto-generated ScienceDirect Webpage on this subject^1^.

An alternative route is to transform the system of nonlinear equations into an operator equation such that every solution to the system is a fixed point of the operator and vice versa. This approach hinges on the premise that the associated operator is a self-mapping from a space into itself. In this paper we investigate the situation when this does not hold and the operator maps one space into a different space. For this scenario we propose an alternating fixed point approach. Specifically, we define a suitable family of fixed point operators that allows constructing an **alternating common fixed points algorithm** that can handle the problem.

However, in order to guarantee the convergence of the algorithm, some quite restrictive conditions on these operators are required. In particular, the approach seems not to be practical for the real-world application in tomographic absorption spectroscopy in which we are interested here.

Facing this kind of problems and motivated by the alternating fixed point approach described above, we take a deeper look at the properties of the equations at hand and suggest to use them in a different way. In particular, we address the case in which the equations depend on two variables, and the dependence on one of them is linear. For this problem, we propose a derivative-free algorithm which also acts alternatingly on each of the variables but makes use of descent directions of the summands of the least squares problem associated to the system of equations. We call it the **descent pairs algorithm** (DPA). The iterative nature of our descent pairs algorithm enables us to introduce a priori conditions into its iterative process. We do this via the **superiorization methodology**. This methodology works by taking an iterative algorithm, investigating its **perturbation resilience**, and then, using proactively such permitted perturbations, it forces the perturbed algorithm to do something useful in addition to what it is originally designed to do. We present a numerical validation of the descent pairs algorithm, with and without superiorization, for a real-world application in tomographic absorption spectroscopy. In this field, problems as the one presented here have been widely studied and a great variety of algorithms have been employed to tackle them. Our experiments show that the approach proposed here yields results that compete well with those obtained by these methods under similar conditions. As a general comment we care to mention that common fixed point iterative methods and related problems are a field of vigorous research with many new directions and developments, see, e.g., [1, 25, 39] and Cegielski’s book [9].

The paper is structured as follows. In Section 2, we briefly recall the well-known fixed point approach for tackling systems of nonlinear equations and present our alternating common fixed points algorithm adapted for the case in which the operator is not a self-mapping. In Section 3, the particular instance of the problem with the linear relation is tackled. We present our descent pairs algorithm and mathematically support the idea behind the algorithmic scheme. Section 4, contains a broad view of tomographic absorption spectroscopy theory and in the last subsection, we include experiments that successfully demonstrate the good performance of our descent pairs algorithm, with and without superiorization, in this field.

## 2. PROBLEM FORMULATION AND AN ALTERNATING COMMON FIXED POINT ALGORITHM

We are interested in solving a system of nonlinear equations as formulated next.

### Problem 1.

Let $R^{M}$ be the Euclidean M-dimensional space. Consider a family of functions $\beta_{k}\;:R^{M}\times R^{M}\to R^{M}$ and vectors $b^{k}={\left(b_{j}^{k}\right)}_{j=1}^{M}\in R^{M}$, for k∈{1,2,...,W}.


(2.1) $$\text{Find}\;x,y\in R^{M}\;\text{such}\;\text{that}\;\beta_{k}\left(x,y\right)=b^{k},\;k=1,\;2,\ldots ,W.$$

The motivation to look at this problem comes from a real-world application in **tomographic absorption spectroscopy** (TAS), see, e.g., Dai et al. [19], that we discuss later in Section 4. In some real-world applications, including TAS, the following condition prevails.

#### *Condition* 1.

No derivatives of the functions $\beta_{k}$ are known and even if they exist they are not calculable.

A common approach for solving systems of nonlinear equations in such a situation consists of translating the system into a **fixed point problem** (FPP), see, e.g., Combettes [18] and Combettes and Pesquet [17].

To illustrate this approach, consider a system of nonlinear equations

(2.2) $$\gamma_{j}\left(z\right)=c_{j},\;j=1,\;2,\ldots ,M.$$

where $\gamma_{j}\;:R^{M}\to R$, for j=1,2,...,M, are given real-valued functions and $c={\left(c_{j}\right)}_{j=1}^{M}\in R^{M}$ is a given vector. Denoting by $\Gamma \;:R^{M}\to R^{M}$ the operator

(2.3) $$\Gamma \;:=\left(\begin{array}{c} \gamma_{1}\\ \gamma_{2}\\ \vdots \\ \gamma_{M} \end{array}\right),$$

the system (2.2) becomes an **operator equation**

(2.4) Γ(z)=c.

Since Γ is a self-mapping, it is well-known how to translate the system (2.2) into a problem of finding a point in the set of fixed points $\mathrm{Fix}\left(T\right)\;:=\left\{x\in R^{M}|T\left(x\right)=x\right\}$ of a suitably defined operator T (see, e.g., Berinde’s book [3, Chapter 8, page 179]). This is done by considering the operator $T\;:R^{M}\to R^{M}$ given by

(2.5) $$T\;:=c+\left(Id-\Gamma \right),$$

where Id : $R^{M}\to R^{M}$ is the identity operator, that is, Id(x)=x. Then, it is easy to see that for a point $x^{*}\in R^{M}$

(2.6) $$T\left(x^{*}\right)=x^{*}\;\text{if}\;\text{and}\;\text{only}\;\text{if}\;\Gamma \left(x^{*}\right)=c$$

and one can solve the system (2.2) by solving the fixed point problem for the operator T.

The difficulty in applying this fixed point approach to the problem posed in (2.1) lies in the fact that $\beta_{k}\;:R^{M}\times R^{M}\to R^{M}$ are not self-mappings. To the best of our knowledge, the challenge of adapting the fixed point theory to this setting has not been attended before. Therefore, we create here an alternating common fixed point algorithm that applies the approach of fixed point theory alternatingly to each of the two vector variables *x* and *y* of the functions $\beta_{k}$ of (2.1).

The adaptation of the fixed point theory to our problem works as follows. For any pair $x,y\in R^{M}$ and for any k=1,2,...,W, define the operators

(2.7) Bkxy:=βk(x,y)βk(x,y).

Then $B_{k}\;:R^{M}\times R^{M}\to R^{M}\times R^{M}$ are self-mappings and, for all k=1,2,...,W,

(2.8) βkx,y=bk⟺Bkxy=bkbk.

Now use the technique of Equation (2.5) and define, for all k=1,2,...,W, the operators

(2.9) Tk≔bkbk+Id-Bk,

i.e.,

(2.10) Tkxy:=bkbk+xy-Bkxy.

Then, for all k=1,2,...,W,

(2.11) Tkx*y*=x*y*⟺βkx*,y*=bk,

and finding a solution to Problem 1 is equivalent to solving the common fixed point problem (CFPP)

(2.12) $$\text{Find}\;\left(\begin{array}{c} x^{\text{*}}\\ y^{\text{*}} \end{array}\right)\in \overset{W}{\underset{k=1}{\cap }}\text{Fix}\left(T_{k}\right).$$

There is an extensive literature of algorithms devoted to solving the CFPP in (2.12), see, e.g., Zaslavski’s book [40]. As an example, the algorithm presented here in Algorithm 1 is the cyclic version of the **almost cyclic sequential algorithm** (ACSA) for the common fixed point problem, in Censor and Segal [14, Algorithm 5], which is, in turn, a special case of the algorithm in Combettes [16, Algorithm 6.1].

For the operators defined in (2.10), Algorithm 1 leads to the proposed **alternating common fixed points algorithm**. It is the iterative process, that starts with an arbitrary x0y0∈RM×RM, and then, for all ℓ≥0, updates according to

(2.13) xℓ+1yℓ+1=xℓyℓ+λℓbi(ℓ)bi(ℓ)-βi(ℓ)xℓ,yℓβi(ℓ)xℓ,yℓ.

However, the convergence theorem for ACSA, see, e.g., [14, Theorem 6], requires additional assumptions on the involved operators. They should be “directed operators”^2^ such that, for all k, $T_{k}-$ Id should be closed at 0. An (uninteresting) example verifying these properties are the projection operators onto the diagonal subspace.

**Algorithm 1:** The Cyclic Sequential Algorithm (CSA) for the common fixed point problem

**1**	**Initialization:** Let ${\left\{T_{k}\;:R^{M}\to R^{M}\right\}}_{k=1}^{W}$ be a finite family of operators. Set ${\left\{\lambda_{\ell }\right\}}_{\ell =0}^{\infty }$ a sequence in [0,2]. Choose $x^{0}\in R^{M}$;
**2**	set ℓ=0;
**3**	**repeat**
**4**	set i(ℓ)=ℓmodW+1;
**5**	set $x^{\ell +1}=x^{\ell }+\lambda_{\ell }\left(T_{i(\ell )}\left(x^{\ell }\right)-x^{\ell }\right)$;

The difficulty of having operators whose block coordinates are identically defined, as is the case for the operators in (2.10), and verifying the assumptions of the convergence theorem for ACSA, lead us to search for an alternative approach to handle the problem. In the next section we develop a different algorithmic scheme which is inspired by the iterative process (2.13).

## 3. THE DESCENT PAIRS ALGORITHM

In this section we propose and motivate a derivative-free algorithm for tackling Problem 1 in the case when the operators fulfill the following two assumptions. We denote the set of vectors whose components are all different from zero by

(3.1) $$R_{\neq 0}^{M}\;:=\left\{x\in R^{M}|x_{j}\neq 0\;\text{for}\;\text{all}\;j=1,\;2,\ldots ,M\right\}.$$

### *Assumption* 1.

For every k∈{1,2,...,W}, the operators $\beta_{k}$ depend linearly on the second variable and nonlinearly on the first variable, such that they can be expressed, in matrix form, as

(3.2) $$\beta_{k}(x,y)=diag\;\left({\tilde{\beta }}_{k}(x)\right)y,\;k=1,\;2,\ldots ,W,$$

where ${\tilde{\beta }}_{k}\;:R^{M}\to R_{\neq 0}^{M}$ and diag(u) denotes the diagonal matrix with the vector u along its diagonal.

For our second assumption, we need to introduce the following definitions.

#### Definition 3.1.

- **Descent direction** (see, e.g., [2, Definition 5.1]). Given a function $g\;:R^{M}\to R$ which is differentiable at some vector $x\in R^{M}$, then a vector $v\in R^{M}$ is called a **descent direction for** *g* **at the point** *x* if
(3.3) $$g^{\prime }(x;v)=\underset{t\to 0}{lim}\;\frac{g\left(x+tv\right)\;-\;g\left(x\right)}{t}=\nabla g{\left(x\right)}^{T}v<0.$$
- **Descent pair**. Given a mapping $f\;:R^{M}\to R^{N}$ and a vector $u\in R^{N}$ we say that (f,u) is a **descent pair** if, for every $x\in R^{M}$, the vector
(3.4) v=v(x):=u-f(x)
  is a descent direction at the point x for the function $g\;:R^{M}\to R$ defined as
(3.5) $$g\left(\cdot \right)\;:=\frac{1}{2}∥u-f\left(\cdot \right){∥}^{2}.$$

### *Assumption* 2.

There exists an index t∈{1,2,...,W} such that $b^{t}\in R_{\neq 0}^{M}$ and such that for all k=1,2,...,W, the pairs $\left(f_{k},u^{k}\right)$, with

(3.6) $$f_{k}(\cdot )\;:=diag\left({\tilde{\beta }}_{t}(\cdot )\right){\tilde{\beta }}_{k}(\cdot )\;\text{and}\;u^{k}\;:=diag{\left(b^{t}\right)}^{-1}b^{k},$$

and where the “−1” power represents matrix inversion, are descent pairs. Moreover, for each $x\in R^{M}$, the pairs $\left(h_{k},b^{k}\right)$, where

(3.7) $$h_{k}(\cdot )\;:=\beta_{k}(x,\cdot ),$$

are descent pairs for all k=1,2,...,W.

In the tomographic absorption spectroscopy problem, modeled as Problem 1 and studied in subsection 4.3 below, Assumptions 1 and 2 are fulfilled.

The next lemma shows that, although Problem 1 is a system which depends on both variables x and y, the Assumption 1 on the operators $\beta_{k}$ implies that the variable vector y does not interfere with the suitability of the variable vector x for solving the system.

### *Lemma* 3.2.

Consider a family of operators $\beta_{k}\;:R^{M}\times R_{\neq 0}^{M}\to R_{\neq 0}^{M}$, for k∈{1,2,...,W}, for which Assumption 1 holds. Then, a point $x^{*}\in R^{M}$ belongs to a solution pair $\left(x^{*},y^{*}\right)$ of the system of equations given by (2.1) if and only if $x^{*}$ is a solution of the system

(3.8) $$diag\;{\left({\tilde{\beta }}_{t}(x)\right)}^{-1}{\tilde{\beta }}_{k}(x)=diag{\left(b^{t}\right)}^{-1}b^{k},\;\text{for}\;\text{all}\;k=1,\;2,\ldots ,W,k\neq t,$$

with t∈{1,2,...,W} such that $b^{t}\in R_{\neq 0}^{M}$.

### Proof.

Let $\left(x^{*},y^{*}\right)$ be a solution of the system given by (2.1). Then, by Assumption 1, for all k,t∈{1,2,...,W}, and using elementary rules for matrix inversion and diagonal matrices, we have

(3.9) $$\begin{array}{l} \beta_{k}\left(x^{*},y^{*}\right)=b^{k}\;\Leftrightarrow diag({\tilde{\beta }}_{k}\left(x^{*}\right))y^{*}=b^{k}\\ \;\Leftrightarrow diag{\left(diag({\tilde{\beta }}_{t}\left(x^{*}\right))y^{*}\right)}^{-1}diag({\tilde{\beta }}_{k}\left(x^{*}\right))y^{*}=diag{\left(b^{t}\right)}^{-1}b^{k}\\ \;\Leftrightarrow diag{\left({\tilde{\beta }}_{t}\left(x^{*}\right)\right)}^{-1}diag{\left(y^{*}\right)}^{-1}diag({\tilde{\beta }}_{k}\left(x^{*}\right))y^{*}=diag{\left(b^{t}\right)}^{-1}b^{k}\\ \;\Leftrightarrow \mathrm{diag}{\left({\tilde{\beta }}_{t}\left(x^{*}\right)\right)}^{-1}{\tilde{\beta }}_{k}\left(x^{*}\right)=\mathrm{diag}{\left(b^{t}\right)}^{-1}b^{k}. \end{array}$$

Thus, $x^{*}$ is a solution for the single vector variable system (3.8). □

In Algorithm 2 below, which we call the **descent pairs algorithm**, we present a method for tackling a system of the form (2.1) that obeys Assumptions 1 and 2. The motivation of the algorithm is as follows. In order to obtain a solution pair $\left(x^{*},y^{*}\right)\;$ to the system (2.1) we generate two separate iterative sequences. The first sequence is denoted by ${\left\{x^{\ell }\right\}}_{\ell =0}^{\infty }$ and employs Lemma 3.2 to find $x^{*}$ as a solution to the system (3.8).

To achieve this, we consider the least squares problem associated with (3.8), which is

(3.10) $$\underset{x\in {\mathbb{R}}^{M}}{\text{argmin}}\sum_{k=1,k\neq t}^{W}g_{k}\left(x\right)=\sum_{k=1,k\neq t}^{W}\frac{1}{2}{\left\|\text{diag}{\left({\tilde{\beta }}_{t}\left(x\right)\right)}^{-1}{\tilde{\beta }}_{k}\left(x\right)-\text{diag}{\left(b^{t}\right)}^{-1}b^{k}\right\|}^{2}.$$

Lines 4 to 8 of the algorithm generate $x^{\ell +1}$ from $x^{\ell }$ by sequentially updating $x^{\ell }$ with constant step-size line searches for each of the summands in (3.10), in the descent direction given by the descent pair provided by Assumption 2.

The purpose of the second sequence ${\left\{y^{\ell }\right\}}_{\ell =0}^{\infty }$ is to find the point $y^{*}$. Lines 9 to 13 show how to obtain $y^{\ell +1}$ from $y^{\ell }$ and $x^{\ell +1}$. Indeed, $y^{\ell }$ is sequentially updated by performing a constant step-size line search for each of the functions

(3.11) $$h_{k}\left(y\right):=\frac{1}{2}{∥b^{k}-\beta_{k}\left(x^{\ell +1},y\right)∥}^{2},\;k=1,\;2,\ldots ,W,$$

in the descent direction determined by the descent pair given by Assumption 2.

**Algorithm 2:** The Descent Pairs Algorithm (DPA).

**1**	**Initialization.** Choose $x^{0},y^{0}\in R^{M}$ Set an index t∈{1,2,…,W} in compliance with Assumption 2 and pick real fixed relaxation parameters $\lambda_{x}>0$ and $\lambda_{y}>0$;
**2**	Set ℓ=0;
**3**	**repeat**
**4**	Set $x^{\ell ,0}=x^{\ell }$;
**5**	**for** q=1,2,…,W **do**
**6**	$x^{\ell ,q}=x^{\ell ,q-1}+\lambda_{x}\left(diag{\left(b^{t}\right)}^{-1}b^{q}-diag{\left({\tilde{\beta }}_{t}\left(x^{\ell ,q-1}\right)\right)}^{-1}{\tilde{\beta }}_{q}\left(x^{\ell ,q-1}\right)\right);$;
**7**	**end**
**8**	Set $x^{\ell +1}=x^{\ell ,W}$;
**9**	Set $y^{\ell ,0}=y^{\ell }$;
**10**	**for** q=1,2,…,W **do**
**11**	$y^{\ell ,q}=y^{\ell ,q-1}+\lambda_{y}\left(b^{q}-\beta_{q}\left(x^{\ell +1},y^{\ell ,q-1}\right)\right)$
**12**	**end**
**13**	Set $y^{\ell +1}=y^{\ell ,W}$;

In Section 4, we illustrate the good performance of the proposed Algorithm 2 in a demonstrative numerical experiment arising from a real-world problem in tomographic absorption spectroscopy, in which our algorithm shows a competitive potential vis-à-vis with state-of-the-art methods in the field.

## 4. AN EXPERIMENTAL DEMONSTRATION IN TOMOGRAPHIC ABSORPTION SPECTROSCOPY

### 4.1. Introduction to tomographic absorption spectroscopy.

Absorption spectroscopy is a popular technique for gas sensing which can simultaneously retrieve thermophysical properties such as temperature, species concentration and pressure [7]. When a laser beam penetrates a **region of interest** (ROI) filled with gaseous medium, its intensity is attenuated due to the absorption of the gas molecules along the **line-of-sight** (LOS).

The aim of absorption spectroscopy is to obtain information of the gaseous medium by measuring spectrum-specified absorbance. The Beer-Lambert law, which relates the attenuation of light to the properties of the material through which the light is traveling, provides the relationship between the gas properties (i.e., pressure, temperature and concentration) and the absorbance, see [33],

(4.1) $$b(v)\;:=ln\left(I_{0}(v)/I_{t}(v)\right)=\int PS(v,x)ydL.$$

The absorbance b(ν) is the logarithmic ratio of the incident $I_{0}(v)$ and transmitted $I_{t}(v)$ intensities for the absorption line at wavelength ν. In (4.1), P is the pressure, which is supposed to be a known constant, and S is the line strength function, whose value depends on the wavelength ν and the temperature x. For each wavelength of interest, the line function S has an approximately negative exponent relation with the reciprocal of the temperature, i.e. 1/x. The concentration of the absorbing species is y and L is the length of the LOS.

**FIGURE 1.**
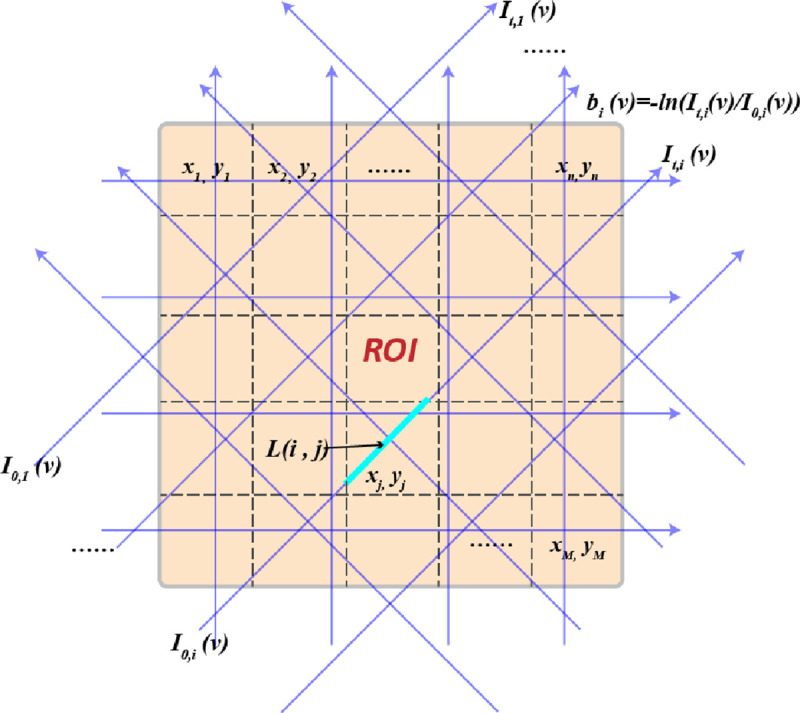
Illustration of the fully-discretized model for tomographic absorption spectroscopy.

In practical applications, the gas properties are usually non-uniform along the LOS, therefore, tomography is needed to enable the spatial resolution of absorption spectroscopy. In order to represent a mathematically tractable model, full discretization is done, meaning that the light spectrum is discretized into a finite number of wavelengths, the ROI is discretized into a finite number of pixels/voxels and the external light sources are discretized into a finite number of individual beams. This modeling of tomographic absorption spectroscopy (TAS) is illustrated in Figure 1.

The discretized model for TAS is obtained as follows. First, the absorbance is measured along a finite number of beams, indexed by i=1,2,...,N, covering the ROI. The light spectrum is discretized into a finite number of wavelengths, indexed by k=1,2,...,W. When N beams are employed and W absorption features are probed in the measurements, the relationship between the temperature x, the concentration of the absorbing species y and the measured absorbance b can be expressed as

(4.2) $$b_{i}^{k}=\int_{L_{i}}\alpha_{v_{k}}\left(x,y\right)dL,k=1,\;2,\ldots ,W,i=1,\;2,\ldots ,N,$$

where $b_{i}^{k}$ is the absorbance along the i-th beam with length $L_{i}$ at the k-th wavelength $v_{k}$, and $\alpha_{v_{k}}$ is the absorption coefficient, related to the wavelength $v_{k}$ and the local values of x and y.

Finally, we assume that the ROI is discretized and consists of a two-dimensional square, meshed into M=n×n square pixels, indexed by j=1,2,...,M. Thus, denoting by $L_{i,j}$ the length of intersection of the i-th light beam within the j-th pixel, equation (4.2) is reinterpreted in its fully-discretized form as

(4.3) $$b_{i}^{k}=\sum_{j=1}^{M}\alpha_{v_{k}}\left(x_{j},y_{j}\right)\cdot L_{i,j}=\sum_{j=1}^{M}\alpha_{k}\left(x_{j},y_{j}\right)\cdot L_{i,j},$$

for k=1,2,…,W, i=1,2,…,N, j=1,2,…,M. The $\alpha_{k\;}:R\times R\to R$ are nonlinear operators which measure the absorption coefficient at the k-th wavelength. Note that after the discretization, x and y are redefined as vectors in $R^{M}$.

Repeating equation (4.3) for all the beams at the *k*-th wavelength, a set of equations is formulated in matrix form as

(4.4) $$b^{k}=\left(\begin{array}{c} b_{1}^{k}\\ b_{2}^{k}\\ \vdots \\ b_{i}^{k}\\ \vdots \\ b_{N}^{k} \end{array}\right)=\left(\begin{array}{ccccc} L_{1,1} & \cdots & L_{1,j} & \cdots & L_{1,M}\\ \vdots & \ddots & \vdots & \ddots & \vdots \\ L_{i,1} & \cdots & L_{i,j} & \cdots & L_{i,M}\\ \vdots & \ddots & \vdots & \ddots & \vdots \\ L_{N,1} & \cdots & L_{N,j} & \cdots & L_{N,M} \end{array}\right)\left(\begin{array}{c} \alpha_{k}\left(x_{1},y_{1}\right)\\ \alpha_{k}\left(x_{2},y_{2}\right)\\ \vdots \\ \alpha_{k}\left(x_{j},y_{j}\right)\\ \vdots \\ \alpha_{k}\left(x_{M},y_{M}\right) \end{array}\right)=L\alpha_{k}(x,y),$$

for all k=1,2,...,W, where L denotes the matrix $L={\left(L_{i,j}\right)}_{i=1,j=1}^{N,M}$. Thus, the fully-discretized modeling of the tomographic problem in TAS results in solving the nonlinear system of equations

(4.5) $$b^{k}=L\alpha_{k}\left(x,y\right),\;k=1,\;2,\ldots ,W,$$

where the vectors $b^{k}$ are known from measurements for all wavelengths and the matrix L is calculated according to the beam arrangement. In the past decades, numerous studies have made progress in this problem. Next, we give a brief glance of it.

### 4.2. Techniques for solving TAS problems.

Both nonlinear or linear approaches have been applied in the literature to solve system (4.5). Depending on the approach for solving the tomographic problem, TAS can be divided into **nonlinear TAS** and **linear TAS**. In nonlinear TAS, all the equation systems are considered together and the inversion is recast into a one-step optimization problem as [30]:

(4.6) $$\underset{x,y\in {\mathbb{R}}^{M}}{\text{argmin}}\frac{1}{2}\sum_{k=1}^{W}{\left\|b^{k}-L\alpha_{k}\left(x,y\right)\right\|}^{2}.$$

The x and y distributions are directly solved from (4.6). This optimization problem is usually solved by a global heuristic optimization algorithm, such as **simulated annealing** (SA) [6, 30].

The SA algorithm was first proposed by Kirkpatrick et al. in 1983 [31] and has been widely employed in large-scale optimization problems [32, 8]. It is a heuristic for finding the global minimum inspired by annealing in metallurgy. A prominent advantage of SA is its insensitivity to the initial guesses [34], while the major drawback is its high computational cost. Besides, a priori information can be taken into consideration [30], which is another advantage of SA.

On the other hand, in linear TAS the problem is divided into two stages. In the first stage, for every k∈{1,2,...,W}, equation (4.4) is solved for each individual wavelength as a linear equation system whose variables are the local absorption coefficients $\alpha_{k}$. Classical algorithms, including **algebraic reconstruction technique** (ART) [26], maximum likelihood expectation maximization (MLEM) [22] and Tikhonov reconstruction [20], have been extensively adopted for this stage.

For the second stage, absorption coefficients $a^{k}={\left(a_{j}^{k}\right)}_{j=1}^{M}\in R^{M}$, at each pixel j and for each wavelength k, are supposed to have been recovered in the first stage, i.e.,

(4.7) $$a^{k}=\left(\begin{array}{c} a_{1}^{k}\\ a_{2}^{k}\\ \vdots \\ a_{j}^{k}\\ \vdots \\ a_{M}^{k} \end{array}\right)=\left(\begin{array}{c} \alpha_{k}\left(x_{1},y_{1}\right)\\ \alpha_{k}\left(x_{2},y_{2}\right)\\ \vdots \\ \alpha_{k}\left(x_{j},y_{j}\right)\\ \vdots \\ \alpha_{k}\left(x_{M},y_{M}\right) \end{array}\right),\;k=1,\;2,\ldots ,W.$$

Now the properties x and y need to be solved according to the nonlinear relationship between them and the absorption coefficients. While, for every k∈{1,2,...,W}, the absorption coefficients $a^{k}$ have been recovered in the first stage, the $\alpha_{k}\;:R\times R\to R$ become here operators. Commonly, in the literature, gas properties within each pixel were calculated from the $a^{k}$ using a **nonlinear fitting** (NF) method [27]. This nonlinear fitting process employs a trust-region-reflective algorithm [15] to find the least-squares of the discrepancy between $a^{k}$ and $\alpha_{k}(x,y)$. The iterative process of this algorithm is briefly described in Algorithm 3, see [35]. In the algorithm, g and H refer to the first and second order derivatives of the function to be solved, respectively, and $s=\left(s_{1},s_{2}\right)$ is the step of the iteration. It should be noted that an analytical expression of the nonlinear tomographic absorption spectroscopy problem is not derivable. Here, the g and H are results of an approximation of the nonlinear tomography formulation.

**Algorithm 3:** The Nonlinear Fitting (NF) method applied in multi-spectral TAS.

**1**	**Initialization:** Set δ∈R. Choose $x_{j}^{0}\geq 0$ and $y_{j}^{0}\geq 0$; set ℓ=0;
**2**	set ℓ=0;
**3**	**repeat**
**4**	set $H=\nabla^{2}({∥a_{j}^{k}-\alpha_{k}(\cdot ,\cdot )∥}^{2})(x_{j}^{\ell },y_{j}^{\ell })$, $g=\nabla ({∥a_{j}^{k}-\alpha_{k}(\cdot ,\cdot )∥}^{2})(x_{j}^{\ell },y_{j}^{\ell })$;
**5**	set $\left(s_{1},s_{2})={argmin}_{∥s∥\leq \delta }(\frac{1}{2}s^{T}Hs+g^{T}s\right)$;
**6**	**if** ${∥a_{j}^{k}-\alpha_{k}(x_{j}^{\ell }+s_{1},y_{j}^{\ell }+s_{2})∥}^{2}<{∥a_{j}^{k}-\alpha_{k}(x_{j}^{\ell },y_{j}^{\ell })∥}^{2}$ **then**
**7**	set $x_{j}^{\ell +1}=x_{j}^{\ell }+s_{1},y_{j}^{\ell +1}=y_{j}^{\ell }+s_{2}$;
**8**	set ℓ=ℓ+1;
**9**	adjust the trust region size δ;
**10**	**else**
**11**	reduce the trust region size δ;
**12**	**end**

A drawback of this method is that it still has a high computational cost. Besides, since the algorithm obtains $x_{j}$ and $y_{j}$ pixel by pixel, a priori information cannot be employed in this algorithm for the process of obtaining temperature and concentration results from absorption coefficients. That means, in traditional approaches for TAS, a priori information is solely employed in the process to solve the absorption coefficients, which is sometimes defective. For example, if the temperature and the concentration satisfy different a priori information, the method to add a priori information on absorption coefficients cannot work. In addition, the calculation of temperature and mole fraction from absorption coefficient can introduce extra errors due to complicated error propagation. In the following sections, we motivate the implementation of our proposed Algorithm 2 for tackling problems of the form given by (4.7), and present a demonstrative example in which it outperforms the NF approach.

### 4.3. Implementation of the descent pairs algorithm.

Let $R_{++}^{M}$ denote the positive orthant of $R^{M}$. For every k∈{1,2,...,W}, denote by $\beta_{k}\;:R_{++}^{M}\times R_{++}^{M}\to R_{++}^{M}$ the operator defined component-wise as

(4.8) $${\left(\beta_{k}(x,y)\right)}_{j}\;:=\alpha_{k}\left(x_{j},y_{j}\right),\;j=1,\;2,\ldots ,M,$$

where $\alpha_{k}\;:R_{++}\times R_{++}\to R_{++}$ are the operators in (4.7). This yields the system of equations

(4.9) $$\beta_{k}\left(x,y\right)=a^{k},\;k=1,\;2,\ldots ,W.$$

It is a known property of the absorption coefficients that the operators $\alpha_{k}$ fulfill Assumption 1 [7]. Thus, there exist operators ${\tilde{\beta }}_{k}\;:R_{++}^{M}\to R_{++}^{M}$ such that

(4.10) $$\beta_{k}(x,y)=diag\;\left({\tilde{\beta }}_{k}(x)\right)y,\;k=1,\;2,\ldots ,W.$$

In order to validate the implementation of the descent pairs algorithm (Algorithm 2) to this problem, we employ a property of the TAS system of equations (4.9) which is known from the physics of the problem and is presented in the next remark.

#### *Remark* 2.

For a fixed index t∈{1,2,...,W}, define for each k∈{1,2,...,W}\{t} the functions

(4.11) $$f_{k}(x)\;:=diag{\left({\tilde{\beta }}_{t}(x)\right)}^{-1}{\tilde{\beta }}_{k}(x).$$

Then, it is known from the physics of the problem that the functions $f_{k}$ are given component-wise by

(4.12) $${\left(f_{k}(x)\right)}_{j}\;:=\frac{S_{k}}{S_{t}}\exp\left(-\left(E_{k}-E_{t}\right)\left(\frac{1}{x_{j}}-\frac{1}{T_{0}}\right)\right),\;j=1,\;2,\ldots ,M,$$

where $S_{k}$, $S_{j}>0$ and $E_{k}$, $E_{t}\in R$ are constants which depend on the wavelength, and $T_{0}$ is a positive known constant.

The following lemmata assure that Assumption 2 holds for the system of equations (4.9).

#### *Lemma* 4.1.

Let t be an index such that $E_{t}=min\;\left\{E_{1},E_{2},\ldots ,E_{W}\right\}$ and let $\overset{‾}{x}\in R_{++}^{M}$ be a fixed arbitrary vector. Then, for any k∈{1,2,...,W}\{t}, the vector given by

(4.13) $$v^{k}\;:=diag{\left(a^{t}\right)}^{-1}a^{k}-f_{k}(\overset{‾}{x})$$

is a descent direction at the point $\overset{‾}{x}$ for the function $g_{k}\;:R_{++}^{M}\to R_{+}$ defined as

(4.14) $$g_{k}(\cdot )\;:=\frac{1}{2}{∥f_{k}(\cdot )-diag{\left(a^{t}\right)}^{-1}a^{k}∥}^{2}.$$

#### Proof.

The partial derivative of the j-th component of $f_{k}$ is given by

(4.15) $$\frac{\partial {\left(f_{k}(x)\right)}_{j}}{\partial x_{\ell }}=\left\{\begin{array}{ll} \frac{S_{k}}{S_{t}}\left(E_{k}-E_{t}\right)\frac{{\left(f_{k}(x)\right)}_{j}}{x_{j}^{2}}, & \text{if}\;\ell =j,\\ 0, & \text{otherwise.} \end{array}\right.$$

Therefore, the gradient of $g_{k}$ at the point $\overset{‾}{x}\in R_{++}^{M}$ is given by

(4.16) $$\nabla g_{k}(\overset{‾}{x})=-\frac{S_{k}}{S_{t}}\left(E_{k}-E_{t}\right)diag\left({\left(\frac{{\left(f_{k}(\overset{‾}{x})\right)}_{j}}{{\overset{‾}{x}}_{j}^{2}}\right)}_{j=1}^{M}\right)v^{k}.$$

Hence, we have

(4.17) $$\nabla g_{k}(\overset{‾}{x}{)}^{T}v^{k}=-\frac{S_{k}}{S_{t}}\left(E_{k}-E_{t}\right)diag\left({\left(\frac{{\left(f_{k}(\overset{‾}{x})\right)}_{j}}{{\overset{‾}{x}}_{j}^{2}}\right)}_{j=1}^{M}\right){∥v^{k}∥}^{2}<0,$$

since $S_{k},S_{t}>0$, $E_{k}-E_{t}>0$ for all k and all diagonal elements of the matrix are positive. □

#### *Lemma* 4.2.

Let $\overset{‾}{x},\overset{‾}{y}\in R_{++}^{M}$. The vector $w^{k}$ given by

(4.18) $$w^{k}\;:=a^{k}-\beta_{k}(\overset{‾}{x},\overset{‾}{y})=a^{k}-diag\left({\tilde{\beta }}_{k}(\overset{‾}{x})\right)\overset{‾}{y}$$

is a descent direction at the point $\overset{‾}{y}$ for the function $h_{k}\;:R_{++}^{M}\to R_{+}$ defined by

(4.19) $$h_{k}(y)\;:=\frac{1}{2}{∥a^{k}-\beta_{k}(\overset{‾}{x},y)∥}^{2}=\frac{1}{2}{∥a^{k}-diag\left({\tilde{\beta }}_{k}(\overset{‾}{x})\right)y∥}^{2},\;\;\text{for}\;\text{all}\;y\in R_{++}^{M}.$$

#### Proof.

The gradient of $h_{k}$ at the point $\overset{‾}{y}$ is given by

(4.20) $$\nabla h_{k}(\overset{‾}{y})=-diag\left({\tilde{\beta }}_{k}(\overset{‾}{x})\right)w^{k}.$$

Thus, we have

(4.21) $$\nabla h_{k}(\overset{‾}{y}{)}^{T}w^{k}=-diag\left({\tilde{\beta }}_{k}(\overset{‾}{x})\right){∥w^{k}∥}^{2}<0,$$

where the last strict inequality holds since ${\tilde{\beta }}_{k\;}:R_{++}^{M}\to R_{++}^{M}$. □

The above analysis shows that the system of equations (4.9) fulfills Assumptions 1 and 2, making Algorithm 2 a good choice for handling it. In Section 4.5 we provide a numerical demonstration of its good performance.

### 4.4. Improving the descent pairs algorithm by superiorization.

The iterative nature of Algorithm 2 enables us to introduce a priori conditions into the iterative process. We do this via the superiorization methodology.

#### 4.4.1. The superiorization methodology.

The superiorization methodology [29] works by taking an iterative algorithm, investigating its **perturbation resilience**, and then, using proactively such permitted perturbations, it forces the perturbed algorithm to do something useful in addition to what it was originally designed to do. The original unperturbed algorithm is called the **basic algorithm** and the perturbed algorithm is called the **superiorized version of the basic algorithm.**

When the basic algorithm is computationally efficient and useful in terms of the application at hand, and the perturbations are simple and not expensive to calculate, then the advantage of this methodology is that, for essentially the computational cost of the basic algorithm, we are able to get something more by steering its iterates according to the perturbations. A detailed description of the superiorization methodology along with pertinent up-to-date references can be found in several papers, see, e.g., [12], [28]

This general principle has been successfully used in a variety of important practical applications, see the recent papers in the, compiled and continuously updated, bibliography of scientific publications on the superiorization methodology and perturbation resilience of algorithms [10], where many applications oriented works with the method appear, e.g., [23].

In the language of [11], the superiorization methodology allows us to employ a given function, called **target function**, $\varphi \;:R^{M}\to R$. It interlaces into the iterations of a basic algorithm steps that perform locally reductions of the target function, these steps are called **perturbations**. The resulting **superiorized version of the basic algorithm** is expected to retain the convergence properties of the basic (unperturbed) algorithm but, additionally, steer the process to an output with reduced, not necessarily minimized, value of the target function.

We use this methodology to superiorize the descent pairs algorithm (Algorithm 2), as we describe below.

#### 4.4.2. Implementation of the superiorized version of the descent pairs algorithm.

In our implementations, priors are applied to Algorithm 2 according to the superiorization methodology. Corresponding target functions φ, mentioned above, to lead the function reduction perturbations in the superiorization process are the priors defined by [38]. These are the **total variation** (TV) function and the **smoothness** (Tikhonov, Tik for short) function [20], defined, respectively, by

(4.22) $$\psi_{TV}(Z):=\sum_{i,j}\sqrt{{\left(Z(i,j)-Z(i+1,j)\right)}^{2}+{\left(Z(i,j)-Z(i,j+1)\right)}^{2}},$$

and

(4.23) $$\psi_{Tik}\left(Z\right):=\sum_{i,j}{\left(Z\left(i,j\right)-\frac{1}{rn}\sum_{ii,jj}\left(Z\left(ii,jj\right)-Z\left(i,j\right)\right)\right)}^{2},$$

where Z represents one of the properties x or y, ${\left\{(i,j)\right\}}_{i=1,j=1}^{n,n}$ is a set of pairs of indices of pixels in an exact grid in the ROI (see Figure 1), rn is the number of pixels that surround pixel (i,j), and (ii,jj) are the indices of these pixels. The pseudo-code of this method applied to (4.9) is shown in Algorithm 4. In the experimental runs the function φ in the algorithm will be either $\psi_{TV}(Z)$ or $\psi_{Tik}(Z)$ of (4.22) or (4.23), respectively. Although in the experiments only the above two functions are chosen as target functions, and thus gradients are involved in the pseudo-code below, it should be emphasized that in general any target function can be considered and function reduction is not necessarily via derivatives [12, 13, 24]. To avoid zero as the divisor when taking gradient for the TV function, we add a small constant 10^−5^ under the square root sign in our application.

In this algorithm, λ is the step length of the DPA iteration and η is the step length for superiorization, which is contracted according to γ as the iteration processes. The value of γ and the initial value of η influence the impact of the superiorization together. We denote by v the normalized gradient of the function φ at the current iteration and z is an intermediate variable between the superiorization and the DPA iteration.

### 4.5. A numerical experiment.

In this subsection, Algorithms 2, 3 and 4 are nicknamed as Algorithm DPA, NF and SUP-DPA, respectively. We conducted simulations on numerous temperature and concentration phantoms, two of which are shown here as demonstrative examples. Phantom 1 mimics the temperature and H_2_O concentration in a two-dimensional McKenna flame. Phantom 2 imitates smooth temperature and concentration distributions consisting of two Gaussian peaks.

The square ROI was divided into 40×40 pixels grids and 160 beams were employed, which were distributed uniformly and parallelly in four directions, 0°, 45°, 90° and 135°. Ten discrete wavelengths were selected from the H_2_O absorption spectrum.

In order to observe the progress and behavior of the DPA algorithm (Algorithm 2) for solving the second stage of the multi-spectral TAS problem, as explained in Subsection 4.2 above, we compared its performance with the NF algorithm (Algorithm 3). In our implementation with MATLAB, the nonlinear least-squares solver, i.e., the function “lsqnonlin”, was applied as the NF algorithm, wherein MATLAB’s trust-region algorithm was employed in this function to find the solution. For the solution of the first stage we employed the superiorized ART algorithm [5, 21].

**Algorithm 4:** The Superiorized version of the descent pairs algorithm (Algorithm 2), nicknamed SUP-DPA, applied in multi-spectral TAS.

**1**	**Initialization:** Set $\lambda_{x},\lambda_{y}>0$, $\eta_{x}$ and $\eta_{y}$. Set the index t such that $E_{t}=min\left\{E_{1},E_{2},\ldots ,E_{W}\right\}$ and choose $x^{0}$ and $y^{0}$ in $R_{+}^{M}$;
**2**	set ℓ=0;
**3**	**repeat**
**4**	set $x^{\ell ,0}=x^{\ell }$;
**5**	**for** q=1,2,…,W **do**
**6**	set $v_{x}:=-\frac{\nabla \varphi \left(x^{\ell ,q-1}\right)}{∥\nabla (\overset{ˆ}{(x,q-1})∥}$;
**7**	**while** $\varphi \left(x^{\ell ,q-1}+\beta_{x}v_{x}\right)>\varphi \left(x^{\ell ,q-1}\right)$ **do**
**8**	set $\eta_{x}=\gamma \eta_{x}$;
**9**	**end**
**10**	set $z_{x}=x^{\ell ,q-1}+\eta_{x}v_{x}$;
**11**	set $x^{\ell ,q}=z_{x}+\lambda_{x}\left(diag{\left(a^{t}\right)}^{-1}a^{q}-diag{\left({\tilde{\beta }}_{t}\left(z_{x}\right)\right)}^{-1}{\tilde{\beta }}_{q}\left(z_{x}\right)\right)$;
**12**	**end**
**13**	set $x^{\ell +1}=x^{\ell ,W}$;
**14**	set $y^{\ell ,0}=y^{\ell }$;
**15**	**for** q=1,2,…,W **do**
**16**	set $v_{y}\;:=-\frac{\nabla \varphi \left(y^{\ell ,q-1}\right)}{∥\nabla \varphi \left(y^{\ell ,q-1}\right)∥}$;
**17**	**while** $\varphi \left(y^{\ell ,q-1}+\beta_{y}v_{y}\right)>\varphi \left(y^{\ell ,q-1}\right)$ **do**
**18**	set $\eta_{y}=\gamma \eta_{y}$;
**19**	**end**
**20**	set $z_{y}=y^{\ell ,q-1}+\eta_{y}v_{y}$;
**21**	set $y^{\ell ,q}=z_{y}+\lambda_{y}\left(a^{q}-\beta_{q}\left(x^{\ell +1},z_{y}\right)\right)$;
**22**	**end**
**23**	set $y^{\ell +1}=y^{\ell ,W}$;

To mimic practical situations, noise was added to the absorbance measurements. Uniform noise was used in our simulations, defined as

(4.24) $$b_{i;mes}^{k}≔b_{i;ori}^{k}\cdot \left(1+rand\cdot u\right),$$

where the subscript ori refers to the original absorbance calculated from the phantom without noise, while mes refers to the practical absorbance with noise added; u is the noise level; rand is a random number in the interval (−1,1).

We tested our algorithms on two different phantoms, Phantom 1 and Phantom 2. For both phantoms, the DPA algorithm was initialized with $\lambda_{x}=\;1000$, $\lambda_{y}\;=\;2$, and $x^{0}$ and $y^{0}$ randomly obtained by picking their components between some lower and upper bounds. These bounds were set, for all j=1,2,...,M, as $x_{j}^{0}\in (400,\;2000)$ and $y_{j}^{0}\in (0.005,\;0.2)$ in the algorithmic runs for the reconstruction of Phantom 1, while for all j=1,2,...,M, $x_{j}^{0}\in (800,\;2400)$ and $y_{j}^{0}\in (0.005,\;0.2)$ in the algorithmic runs for the reconstruction of Phantom 2.

For the SUP-DPA algorithm (Algorithm 4), in the reconstruction of Phantom 1, TV was employed as the prior, i.e., as the function φ in the algorithm. The SUP-DPA algorithm was initialized with $\lambda_{x}=1000$, $\lambda_{y}=2$, $\beta_{x}=5\times 10^{6}$, $\beta_{y}=10$ and γ=0.999. Initial guesses of $x^{0}$ and $y^{0}$ were vectors randomly chosen in the intervals described above for the DPA algorithm.

For the reconstruction of Phantom 2 with the SUP-DPA algorithm, smoothness was regarded as the prior, thus, the function φ in the algorithm was chosen as *ψ*_*Tik*_ of (4.23) and the algorithm was initialized with $\lambda_{x}=1000$, $\lambda_{y}=2$, $\beta_{x}=5\times 10^{4}$, $\beta_{y}=10$ and γ=0.999. Initial guesses of $x^{0}$ and $y^{0}$ were vectors randomly picked in the intervals described above for the DPA algorithm.

**FIGURE 2.**
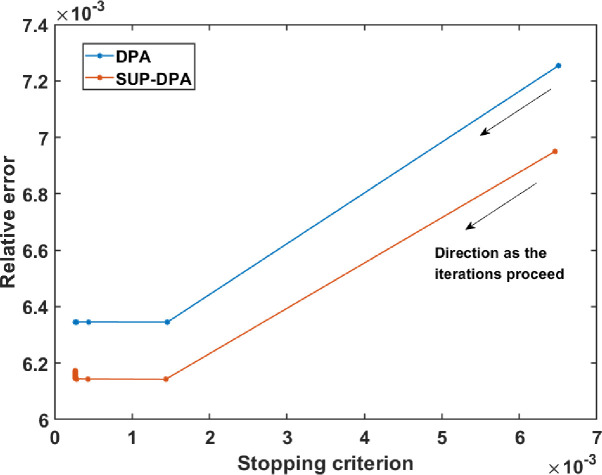
Relation between relative error and stopping criterion as the iterations proceed.

All runs of the DPA and the SUP-DPA algorithms were stopped when either the number of iterations exceeded 50 or when $\sum_{k=1}^{W}\;∥b^{k}-\beta_{k}\left(x^{\ell +1},y^{\ell +1}\right)∥<10^{-3}$. Figure 2 shows the relation between relative error in x and the stopping criterion function $\sum_{k=1}^{W}\;∥b^{k}-\beta_{k}\left(x^{\ell +1},y^{\ell +1}\right)∥$ as the iterations proceed for DPA and SUP-DPA. The relative errors are defined as

(4.25) $$\left\{\begin{array}{l} {Error}_{x}≔\frac{{∥x_{rec}-x_{act}∥}_{2}}{∥x_{act}{∥}_{2}},\\ {Error}_{y}≔\frac{{∥y_{rec}-y_{act}∥}_{2}}{{∥y_{act}∥}_{2}}, \end{array}\right.$$

where the subscripts “rec” and “act” stand for reconstructed value and actual value, respectively.

It can be seen that after the stopping criterion function value fell below 10^−3^, the relative error hardly changes, indicating that a convergence is reached. The relative error of SUP-DPA had a slight fluctuation at the left end of the curve because of the perturbation introduced. On the other hand, 50 iterations is a large enough number for this testing case to assume convergence when the stopping criterion function value cannot decrease to 10^−3^. For the NF algorithm, the function tolerance and optimality tolerance were both set to 5×10^−10^. When implementing the NF algorithm, δ was initially set as the 2-norm of the difference between the upper and lower bounds, ((800,2400) for x and (0.005,0.2) for y), and the adjustment of δ followed the default settings in the MATLAB function “lsqnonlin”. The algorithm was terminated when $\delta <5\times 10^{-10}$.

**FIGURE 3.**
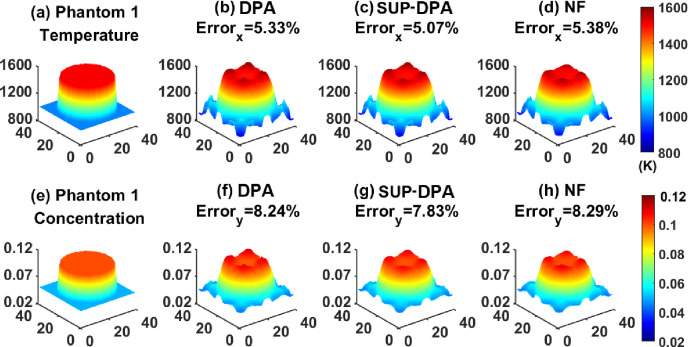
(a) Temperature profile of Phantom 1. Temperature profiles recovered by (b) the DPA algorithm, (c) the SUP-DPA algorithm, and (d) the NF algorithm. (e) Concentration profile of Phantom 1. Concentration profiles recovered by (f) the DPA algorithm, (g) the SUP-DPA algorithm, and (h) the NF algorithm.

**FIGURE 4.**
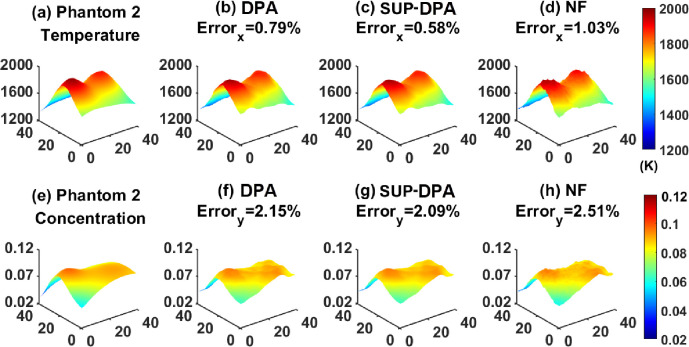
(a) Temperature profile of Phantom 2. Temperature profiles recovered by (b) the DPA algorithm, (c) the SUP-DPA algorithm, and (d) the NF algorithm. (e) Concentration profile of Phantom 2. Concentration profiles recovered by (f) the DPA algorithm, (g) the SUP-DPA algorithm, and (h) the NF algorithm.

Results are shown in Figures 3 and 4 and Table 1. The name of the algorithms is shown at the top of the corresponding reconstructed profiles. The relative reconstruction errors are also labeled, which are defined by (4.25). To be specific, the unit of temperature x was K, which is labeled beneath its color-bar in the figures, and for the mole fraction y, the unit is one, thus, no unit was labeled.

Figure 3 shows Phantom 1 and the results recovered by the DPA, the SUP-DPA and the NF algorithms. Temperature and concentration profiles generated by the three algorithms show similar shapes to those of Phantom 1, although there are some defects at the edges of the profiles. The reconstruction errors of SUP-DPA is smaller than those of the DPA and the NF algorithm. Besides, it takes much less time for the DPA and the SUP-DPA algorithms to finish the reconstruction than it took for the NF algorithm. Overall, the SUP-DPA algorithm can be regarded as the best among the three algorithms, for the particular experiments that we conducted and report here.

Figure 4 shows Phantom 2 and the results recovered by the DPA, the SUP-DPA and the NF algorithms. Results by the three methods show a similar shape to Phantom 2.

The error of the DPA algorithm is slightly smaller than that of the NF algorithm, while the SUP-DPA algorithm improves the error of both other algorithms. Besides, the computational time of the SUP-DPA algorithm is much smaller than that of the NF algorithm. Though the DPA algorithm has the lowest computation time, its reconstruction effect is worse than that of the SUP-DPA algorithm. Therefore, the SUP-DPA algorithm can still be regarded advantageous over the DPA and the NF algorithms.

**FIGURE 5.**
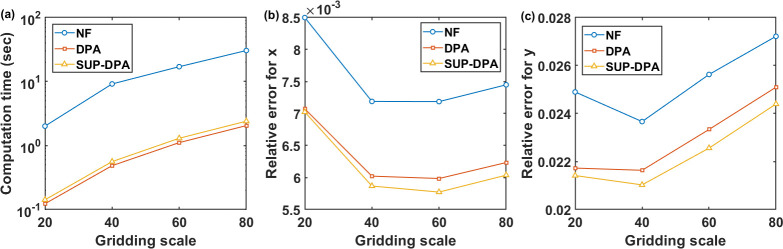
(a) Computation time, (b) reconstruction error for x, and (c) reconstruction error for y, with respect to the gridding scale.

To further investigate the performance of the algorithms, simulation studies were conducted under different spatial resolutions. For Phantom 2, reconstructions were conducted when it was divided into 20 × 20, 40 × 40, 60 × 60 and 80 × 80 pixel grids, denoted as “gridding scale” 20, 40, 60 and 80, respectively. To ensure the comparability of the results, the measurement of each case was conducted in four directions, while the number of beams in each direction increased correspondingly to the gridding scale, which means that for a *G*×*G* grid, 4×*G* measurement beams were applied. Other conditions, including noise level, parameters and stopping criteria, were the same as those described in Figure 4. The results are shown in Figure 5.

As the pixel grid was made finer, the computation time grew exponentially, indicating an increasing computational efficiency improvement of DPA and SUP-DPA compared with NF. In each simulation condition, DPA and SUP-DPA had better performance than NF, with SUP-DPA smaller than DPA in relative error and DPA slightly smaller in computation time. At gridding scale 20, there were the largest errors. This might be due to less measurement beams. Coarser gridding made the phantom less smooth, which could result in difficulties for the reconstruction.

## Figures and Tables

**Table 1.** Computation times of the algorithms

Computation time (sec)	DPA	SUP-DPA	NF
Phantom 1	0.48	0.56	9.16
Phantom 2	0.48	0.54	7.67

## Footnotes

1 https://www.sciencedirect.com/topics/engineering/simulated-annealing-algorithm.

2 Directed operators are nowadays called “cutters”, see [9, Subsection 2.1.3]
